# Does Mechanocrine Signaling by Liver Sinusoidal Endothelial Cells Offer New Opportunities for the Development of Anti-fibrotics?

**DOI:** 10.3389/fmed.2019.00312

**Published:** 2020-01-09

**Authors:** Sumeyye Soydemir, Olivia Comella, Dina Abdelmottaleb, James Pritchett

**Affiliations:** ^1^Department of Life Sciences, Faculty of Science and Engineering, Manchester Metropolitan University, Manchester, United Kingdom; ^2^Centre for Bioscience, Faculty of Science and Engineering, Manchester Metropolitan University, Manchester, United Kingdom

**Keywords:** liver, endothelial, LSEC, PIEZO, HSC, fibrosis, ECM, mechanocrine

## Introduction

**Figure 1:**
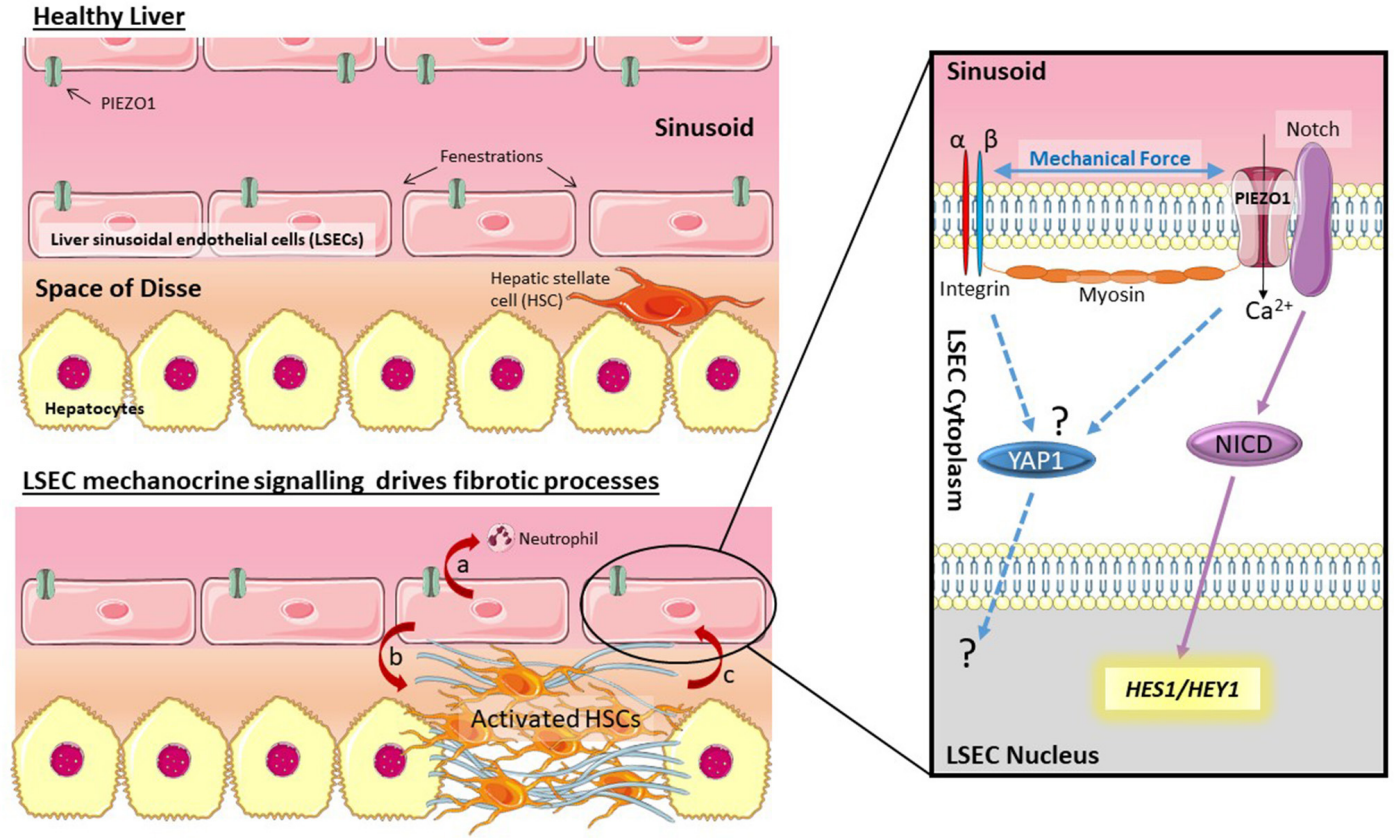
Mechano-sensing by LSECs drives fibrotic processes. LSECs can respond to changes in shear stress and pressure in the sinusoid through activation of PIEZO channels. Data by Hilscher et al. (4) suggests this is triggered by integrins and myosin filaments. PIEZO channel activation drives cleavage of Notch to release NICD, and transcription of Notch pathway genes *HES1* and *HEY1*. Activation of this mechanism results in chemokine secretion (CXCL1) which recruits neutrophils (a). Signaling by LSECs is also known to trigger HSC activation (b) which leads to stiffening of the ECM, potentially driving activation of other mechano-sensitive pathways (c) such as YAP1.

Liver sinusoidal endothelial cells (LSECs) are specialized endothelial cells that have essential roles in normal liver homeostasis, and are also involved in disease processes. The importance of LSEC biology has recently been extensively reviewed (1, 2). LSECs line the walls of the hepatic sinusoid (Figure 1) where they scavenge blood borne macromolecules. LSECs are constantly exposed to antigens carried from the gastrointestinal tract by the portal vein. LSECs therefore have a crucial role, alongside Kupffer cells, as gate keepers for liver immunomodulation. If LSEC immune responses are dysregulated, the result is chronic inflammation which can drive the development of fibrosis (2).

LSECs maintain a perforated plasma membrane to form fenestrations ranging between 50 and 300 nm in diameter (3). In a healthy, functioning liver, blood enters the sinusoids via the portal vein and hepatic artery, thus enabling oxygen and macromolecules to be transferred across the endothelial barrier to hepatocytes, facilitated by the LSEC fenestrae (1).

Due to their location lining the sinusoid LSECs (Figure 1) are in direct contact with blood flow and therefore exposed to changes in both shear stress and blood pressure. Numerous researchers have made this observation, however recent reviews of LSEC biology (1, 2) also illustrate how little is known about mechano-sensing pathways in LSECs. A recent article by Hilscher et al. (4) has now highlighted how mechano-sensitive pathways in LSECs can drive recruitment of circulating blood cells to drive portal hypertension. Mechanocrine signaling by LSECs can orchestrate complex responses across cell types and tissues. This article will highlight the importance of mechano-biology in LSECs during liver disease and point out important gaps in knowledge. This exciting research topic has the potential to reveal novel targets for the development of urgently needed anti-fibrotics.

Importantly LSECs are able to modulate phenotypic changes in hepatic stellate cells (HSCs) (5–7). HSCs are responsible for the altered extracellular matrix (ECM) production characteristic of liver fibrosis (8). In the healthy liver HSCs reside in the space of disse between the endothelial (LSEC) layer and epithelial (hepatocyte) layer. In response to fibrogenic cues, including inflammatory signals from hepatocytes or LSECs, HSCs alter their phenotype to become activated myofibroblasts. Activated HSCs are proliferative, migratory, and contractile cells that secrete fibrotic ECM (9). This means that mechanically induced changes in LSECs have the potential to rapidly alter HSC phenotype and drive fibrogenesis. The fact that LSEC dysfunction precedes the development of fibrosis in non-alcoholic liver disease (10) supports the hypothesis that signals from LSECs may be one of the earliest triggers of HSC activation. There is also the potential for the establishment of a positive feedback loop in which mechanically activated LSECs trigger mechanocrine signaling that activates HSCs. In turn, activated HSCs alter the ECM to increase tissue stiffness, driving further mechano-activation of both LSECs and HSCs. Drugs that in some way break this mechanocrine feedback loop could have great therapeutic potential for the treatment of fibrotic disease.

## Mechano-Biology in Liver Disease

Key experiments by Rebecca Wells's group clearly showed that liver stiffness changes very early following hepatic injury (11), and that increased substrate stiffness is necessary for HSC activation (12, 13), a key step in fibrogenesis This raises the question of whether increased hepatic stiffness is a symptom or a driver of liver disease. Or both? Mechanical force across a tissue can change due to fluctuations in blood pressure, the behavior of contractile cells (e.g., HSCs) and changes in the ECM. Following liver injury changes in hepatic blood pressure occur rapidly (11, 14), and hypertension in the context of non-alcoholic fatty liver disease appears to increase the risk of fibrosis (15, 16).

Interest in mechano-sensing during fibrotic liver disease has largely focused on HSCs (12, 17, 18), and several mechanically sensitive signaling pathways have been shown to function in HSCs. Latent TGFbeta, a pro-fibrotic cytokine (19), is released from the ECM by contractile force transmitted from HSCs via the α_v_ integrin subunit (20). Furthermore, the mechano-sensitive transcriptional regulator Yes Associated Protein 1 (YAP1) (21) is activated in HSCs by increased substrate stiffness (22, 23). YAP1 can be inhibited using verteporfin (24) to reduce fibrosis *in vivo* (23). By contrast, relatively little is known about how LSECs sense and respond to external mechanical cues.

### Portal Hypertension and Regulation of Sinusoidal Tone

Changes in vascular tone cause rapid changes in blood pressure, shear forces and the overall mechanical stiffness of the liver (14). LSECs regulate vascular tone by releasing vasoconstrictors, e.g., cyclooxygenase 1 (COX1) and thromboxane A2 (TXA2); and vasodilators, e.g., NO which act on HSCs to modulate their contraction and therefore regulate sinusoidal pressure (25). Some studies suggest that endothelin, a potent vasoconstrictor, has an important role in driving portal hypertension, as patients with cirrhosis have an increased circulating ET-1 (26). When liver injury occurs, HSCs secrete Endothelin-1 (ET-1), establishing an autocrine loop contributing to increased blood pressure (14, 27, 28). Intriguingly, recent data suggests that ET-1 activates YAP-1 in ovarian cancer cells (29). Tocci and co-workers showed that beta-arrestin, functioning downstream of ET_A_R, physically interacts with YAP1 to increase nuclear shuttling.

Research is now beginning to reveal how LSECs detect and respond to changes in hepatic blood flow and altered ECM stiffness.

## Potential for Mechano-Signaling by LSECs

LSECs are exposed to mechanical cues derived from both blood flow/pressure changes and changes in the surrounding ECM of the liver during fibrotic disease. Endothelial cell populations in other vascular beds are able to detect and respond to mechanical cues, so it seems reasonable to suggest similar mechanisms would exist in LSECs. Several different mechano-signaling pathways, including Neurogenic locus notch homolog (Notch) 1 (30), PIEZO channels (31–33) and YAP1 (34), have all been shown to function in endothelial cells. Furthermore, as described above, ET-1 can drive YAP1 nuclear shuttling (29). This makes possible a positive feedback loop where HSCs activated by mechanical cues release ET-1, which could have a dual function. (1) Autocrine constriction of activated HSCs, contributing to portal hypertension and increased liver stiffness; and (2) YAP1 activation in both HSCs and LSECs, due to ET-1 signaling, *and* increased mechanical stiffness.

### Notch

Notch proteins are transmembrane proteins that undergo proteolytic cleavage upon ligand binding. Notch ligands are themselves membrane bound proteins from the jagged and delta families. Upon binding to jagged or delta proteins presented by neighboring cells, Notch proteins are cleaved to release an intracellular domain (NICD) that translocates to the nucleus to orchestrate transcriptional regulation (35). This highly conserved mechanism allows cell-to-cell contact to regulate key processes such as proliferation, cell fate, differentiation, and cell death.

Notch proteins are expressed by vascular endothelial cells (36), and play a critical role in development of the vascular system (37). Mechanical force is necessary to reveal the Notch cleavage site and allow release of NICD (38, 39). It has recently been shown that Notch1 localization in endothelial cells is polarized by shear force. Notch1 protein polarization occurs in the direction of flow, and Notch1 is aligned with the downstream direction of flow across the endothelial cell layer (30). Furthermore, levels of nuclear NICD increased in a step wise fashion as shear stress induced by flow increased, providing compelling evidence that endothelial Notch is a mechano-sensor (30) that regulates endothelial function and phenotype in response to changes in shear stress.

In the liver Notch is expressed by LSECs (40, 41). Targeted deletion of *Notch1*, or the canonical notch effector *Rbpj1*, specifically in LSECs, caused dilated sinusoids and portal hypertension in adult mice (42). When Notch1 protein expression was disrupted in LSECs at birth, development of the liver vasculature was severely disrupted (42). Conversely, forced Notch pathway activation by endothelial specific overexpression of NICD also disrupted normal liver homeostasis, with expanded sinusoids, reduced hepatocyte proliferation and increased hepatocyte cell death. LSECs appeared to become dedifferentiated, and the fibrogenic response to CCl4 induced liver injury was increased (43).

These findings highlight the importance of tightly regulated Notch1 signaling in LSECs for normal liver function. Mechanical regulation of Notch1 could play a critical role in normal liver homeostasis, and in the response to liver injury. Intriguingly, recent data (4) shows that the Notch1 pathway in LSECs is sensitive to mechanical cues. Hilscher et al. (4) suggest that stretch activated PIEZO cation channels activate Notch signaling which drives recruitment of neutrophils and formation of neutrophil extracellular traps that cause portal hypertension.

### PIEZO Channels

PIEZO proteins form mechano-sensitive cation channels in the plasma membrane (44, 45). PIEZO1 is essential for correct vascular development, and global knockout of *PIEZO1* is lethal (31, 32). PIEZO1 channels are present in the plasma membrane of endothelial cells and activated by shear stress to trigger Calcium influx into the cell (31, 32). Since their initial discovery, it has been shown that PIEZO1 is also critical for normal vascular homeostasis. Endothelial cells respond to changes in shear forces via PIEZO1. PIEZO1 induced signaling elicits downstream changes in vascular tone and blood pressure. In mice with endothelial specific PIEZO1 deficiency the ability of endothelial cells to respond to changes in flow by releasing NO to trigger vasodilation was lost, resulting in hypertension (33).

PIEZO channels are present on LSECs (31), and, as mentioned above, Hilscher et al. have recently highlighted how PIEZO1 channels modulate Notch pathway activity in response to changes in blood pressure (4). In their experimental model of cyclic stretch, integrins transmitted changes in mechanical force to activate PIEZO1 cation channels, possibly via myosin (46, 47). Similarly, force transmitted via non-muscle myosin has recently been shown to be involved in the ligand-activated cleavage of Notch (48). In LSECs the integrin-activated PIEZO1 channels interact with the Notch1 receptor to activate Notch target genes via production of the transcription factors Hes1 and Hey1 (4). Future experiments are necessary to establish whether myosin filaments in LSECs can interact directly with Notch1, or via PIEZO1, to drive notch cleavage and downstream signaling. It is also important to note that the actomyosin cytoskeleton has a crucial role in maintaining the fenestrated plasma membrane characteristic of healthy LSECs (49–51). This adds further complexity to the interplay between external and internal mechanical forces. How are changes in external force transmitted into LSECs? How do changes in external force affect the LSEC cytoskeleton? Could external mechanical cues have a direct influence on the maintenance of the fenestrated plasma membrane?

### YAP1

Another mechanism for mechano-signaling in LSECs is YAP1, which has recently been shown to be sensitive to shear forces in zebrafish endothelial cells (34). Nuclear YAP1 is also present in primary LSECs isolated from murine livers (52). YAP1 can be activated downstream of PIEZO1 (46). Further work is therefore necessary to confirm YAP1 expression and function in mammalian LSECs, and whether YAP1 status in LSECs can be regulated by PIEZO channel activation. Current understanding of YAP1 function in the liver has recently been extensively reviewed (53).

## Therapeutic Potential

LSEC phenotype restoration through inhibition of mechano-sensitive pathways provides an intriguing therapeautic strategy for the treatment, and even reversal, of liver fibrosis. Compelling evidence that LSECs signal to neighboring cells in a context dependent manner to drive either tissue regeneration or fibrosis (7) provides strong support for the targeting of LSECs as a means to drive fibrosis regression. As many of the pathways discussed are not specific to LSECs, or even to endothelial cells, a means of delivering a therapy specifically to LSECs is desirable. Nano-particles targeting LSECs for the regulation of auto-immunity have already been developed (54). Similar approaches could be used to deliver molecules targeting mechano-sensing pathways specifically to LSECs. Timing of therapy will be crucial. Early intervention would arguably provide more chance of success, but this is made challenging due to issues with late diagnosis. However, clearance of hepatitis C infection leads to fibrosis regression, and clearly shows that human liver fibrosis is reversible at later stages than previously thought (55).

### Targeting Notch

Two classes of drug that target notch signaling are currently in clinical trials as cancer therapies (56). (1) Gamma-secretase inhibitors (GSIs) target the enzymes responsible for cleavage of Notch and block release of NICD. (2) Monoclonal antibodies block notch-ligand receptor interactions. Both classes of drug have dose limiting side effects linked to normal notch function in the gastrointestinal tract. Successful adoption of notch inhibition as a therapeutic strategy for liver fibrosis would therefore require cellular targeting to avoid severe side effects. As mentioned previously (section NOTCH), Notch has diverse functions during liver development, homeostasis and disease (57). In hepatocytes (58) or LSECs (43) Notch signaling can induce HSC activation and promotes fibrosis. It has been demonstrated that inhibition of Notch signaling using a GSI *in vivo* ameliorated fibrosis in a CCl4 pre-clinical model (59). Therefore, therapeutic targeting of Notch would impact multiple pro-fibrotic mechanisms, potentially including mechano-crine signaling by LSECs (4).

### Targeting PIEZO Channels

Yoda1 was the first molecule identified which could artificially regulate PIEZO channel activity (60). However, Yoda1 functions as an agonist and causes activation of PIEZO1. Based on the evidence from Hilscher et al. activating PIEZO1 would have a negative impact on liver fibrosis (4). Dooku is a more recently identified analog of Yoda1, which appears to function as a Yoda1 antagonist (61). Importantly this molecule only inhibits Yoda1 induced PIEZO channel activation. As yet, no small molecule antagonists of PIEZO channel mechano-activation have been discovered. It is interesting to speculate what effect PIEZO channel inhibitors might have on liver fibrosis, especially if they could be delivered specifically to LSECs. As PIEZO receptors are widely expressed across endothelial cell types, long term global treatment with a PIEZO antagonist would likely have undesirable side effects.

### Integrins

Hilscher et al. demonstrate that PIEZO channel mechano-activation is triggered by integrin signaling; treatment of cells with arginine-glycine-aspartate (RGD) peptide inhibited stretch-induced transcription of Notch target genes (4). Identification and targeting of the integrin heterodimers (62) involved in this mechanism could be a strategy for developing anti-fibrotics. The integrin subunits present in the LSEC cell membrane are yet to be fully characterized. Mass spectrometry showed that integrin beta 3 is expressed by LSECs following partial hepatectomy (63). Candidate integrin alpha subunits include alphaV and alphaIIb, both of which partner with the beta3 subunit to facilitate interactions between LSECs and platelets (64).

### Targeting YAP1?

Verteporfin (tradename Visudyne, Novartis) was originally developed as a light activated treatment for neovascular macular degeneration (65). Verteporfin's ability to inhibit YAP1 activity was identified by screening for compounds able to disrupt the interaction between YAP-1 and it's DNA binding partner TEAD1 (24). Mice tolerate verteporfin treatment via intraperitoneal injection over 3 weeks (23). However, further studies are needed to assess its specificity and potential for development as a long term therapeutic strategy. In light of this it is important to note that more specific alternatives to verteporfin have already been developed and tested *in vitro* (66).

## Discussion

The data presented by Hilscher et al. (4) is compelling: mechanical cues alter LSEC function. In response to mechanical stretch PIEZO channels activate the notch pathway to trigger secretion of the chemokine CXCL1 by LSECs. CXCL1 release recruits neutrophils that drive microthrombi formation and promote portal hypertension. This is the first direct evidence of mechano-sensing by LSECs, and links PIEZO channels with notch-signaling, both of which are known to be mechanically activated in other contexts. It is reasonable to expect that integrins will also be involved in the detection of mechanical cues by LSECs. For other mechanosensitive pathways such as YAP/TAZ there is potential for involvement in LSEC biology as YAP1 responds to shear stress in a zebrafish model (34). Another area of interest is how actomyosin contractility responds to and generates force to regulate LSEC shape (fenestrae) and integrate external and internal cues via PIEZO (47), notch (48), or YAP1 (67). The next challenge will be to harness our improving understanding of the importance of mechanobiology in LSECs to attempt to develop novel therapies for liver disease. Breaking the positive feedback loop set in motion when mechanical cues cause LSECs to trigger neutrophil recruitment, and potentially HSC activation, could be a successful therapeutic strategy.

## Author Contributions

SS and OC researched the topic and prepared draft text and figure. DA edited the text and provided feedback. JP supervised, SS and OC managed the preparation of the manuscript, researched the topic, and prepared the final text.

### Conflict of Interest

The authors declare that the research was conducted in the absence of any commercial or financial relationships that could be construed as a potential conflict of interest.
